# More than just noise: Chance, mating success, and sexual selection

**DOI:** 10.1002/ece3.7484

**Published:** 2021-03-30

**Authors:** Hope Klug, Libby Stone

**Affiliations:** ^1^ Department of Biology, Geology, and Environmental Science University of Tennessee at Chattanooga Chattanooga TN USA; ^2^ SimCenter University of Tennessee at Chattanooga Chattanooga TN USA; ^3^ The Honors College University of Tennessee at Chattanooga Chattanooga TN USA

**Keywords:** chance, mate competition, mate limitation, operational sex ratio, population size, sexual selection

## Abstract

Chance plays a critical but underappreciated role in determining mating success. In many cases, we tend to think of chance as background noise that can be ignored in studies of mating dynamics. When the influence of chance is consistent across contexts, chance can be thought of as background noise; in other cases, however, the impact of chance on mating success can influence our understanding of how mates are acquired and how sexual selection operates. In particular, when the importance of chance covaries with biological or ecological factors in a systematic manner—that is, when chance becomes consistently more or less important under certain conditions—then chance is important to consider if we want to fully understand the operation of mate acquisition and sexual selection. Here, we present a model that explores how chance covaries with factors such as sex ratio, adult population size, and mating regime in determining variation in mating success. We find that in some cases, chance covaries with adult population size and the operational sex ratio to create variation in mating success. We discuss how chance can influence our more general understanding of the operation of mating dynamics and sexual selection.

## Graphical abstract

The effect of chance on mating success can covary with biological factors of interest and influence our understanding of sexual selection. In such cases, chance becomes more than just noise, and considering chance is key to understanding fully the operation of sexual selection.

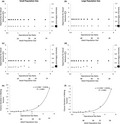

## 1 INTRODUCTION

Understanding the relative role that sexual selection plays in the evolution of traits is critical to understanding biodiversity (Edward & Chapman, 2011; Fromhage & Jennions, 2016; Harrison et al., 2015; Kokko & Rankin, 2006; Liker et al., 2013, 2015; Liker & Székely, 2005; Lumley et al., 2015; Servedio & Boughman, 2017; Tobias et al., 2012; Wagner et al., 2012) and requires knowledge of the conditions under which sexual selection will be more or less important relative to other factors in determining the total selection on traits. For example, sexual selection and natural selection often interact to influence mating and reproductive success, selective regimes, and trait evolution and can vary in their relative importance (Cornwallis & Uller, 2010; House et al., 2013; Mann & Seehausen, 2011). In addition to natural selection, chance plays a critical but underappreciated role in determining mating success (Gowaty & Hubbell, 2005, 2009, 2013; Hubbell & Johnson, 1987; Jennions et al., 2012; Sousa & Westneat, 2013; Sutherland, 1985, 1987) and can affect when sexual selection will be relatively strong versus weak. In some cases, the effect of chance on mating success is obvious. For example, if, by chance, a given environment makes mate assessment relatively difficult, sexual selection on a preferred trait would be relatively weak compared to an environment that allows for easy mate assessment. In such a simplistic case, the effect of chance on sexual selection is relatively straightforward: We would expect chance to decrease the strength of sexual selection because trait values will determine relatively less variation in mating success than a scenario in which mate assessment is relatively easy. In other cases, however, the effect of chance on mating success is less intuitive, particularly when the effect of chance varies systematically with other factors, such as sex ratio. In such cases, it becomes difficult to predict a priori how various factors, such as sex ratio, will influence sexual selection (Jennions et al., 2012).

Some previous research has noted the importance that chance events can have in determining mating success. For example, studies have found no evidence of mate preference or sexual selection in rats (*Rattus norvegicus*) (Le Moëne & Snoeren, 2018) and red‐winged blackbirds (*Agelaius phoeniceus*) (Westneat, 2006) under some conditions, suggesting a potential role for chance in determining mating success. Likewise, other researchers have highlighted the role that random factors play in determining mating success (Gowaty & Hubbell, 2005, 2009; Gowaty et al., 2012; Hubbell & Johnson, 1987; Snyder & Gowaty, 2007; Sutherland, 1987; Tang‐Martinez & Ryder, 2005). For example, Kokko and Mappes illustrated that chance events influencing mating encounters can affect mating dynamics (Kokko & Mappes, 2013), and Sutherland (Sutherland, 1985) and Hubbell and Johnson (Hubbell & Johnson, 1987) noted that even when there is evidence of sexual selection, chance can still lead to variation in mating success that is not attributable to sexual selection. Some studies have even suggested that the sex differences in variance in mating success observed in Bateman's classic study (Bateman, 1948) can be explained by random mating rather than male–male competition (Gowaty et al., 2012, 2013; Sutherland, 1985).

Consistent sex roles cannot be caused by chance alone, and there is substantial evidence for nonrandom mating patterns across animals (Schärer et al., 2012) (but see (Ah‐King, 2013; Kokko et al., 2013) for further discussion). However, chance clearly influences mating dynamics. Despite its potential importance in understanding the operation of mate acquisition, chance is typically ignored in most studies of sexual selection and treated as if it is simply “noise.” The few studies that focus on chance typically do not focus on covariation between chance and other factors of interest. As a result, incredibly little is known about how and when chance affects sexual selection, and more importantly, how any effects of chance covary with other factors of biological interest (e.g., parental investment, density, population size, mate sampling strategies) in determining mating success. When the importance of chance covaries with biologically relevant factors in a systematic manner, chance becomes more than simply background noise; that is, if chance predictably varies with factors such as sex ratio and mate sampling regime, understanding the influence of chance on mating success across various scenarios is key to understanding the relative role of sexual selection in evolutionary dynamics. Indeed, understanding when chance covaries with biological factors of interest is essential for predicting the conditions under which we would expect sexual selection to be relatively more or less important in shaping evolution.

**FIGURE 1:**
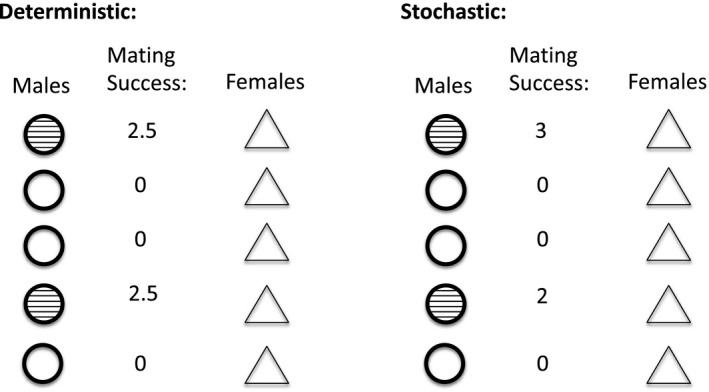
Chance variation in mating success occurs due to the fact that mate numbers must be an integer. There is often chance variation in mating success due to the fact that mate number must be an integer. Here, we depict a population of five males (represented as circles) and five females (represented as triangles) in which two of the males have a sexually selected trait that allows them to acquire mates (represented as stripes). This trait could be preferred in mate choice or give males an advantage in male–male competition. We assume that males can mate multiply, but females can mate only once. If mating was purely deterministic and two males had the preferred trait, each of the two males with the preferred trait would have a mating success of 2.5. In such a case, the strength of sexual selection (i.e., the selection differential) would be 0.6 and the opportunity for sexual selection, a standardized measure of the variation in mating success, would be 1.5. However, because mate number must be an integer, there is stochasticity in mating. If we account for the fact that mate number must be an integer, one of the males with the preferred trait will have a mating success of 3, whereas the other male will have a mating success of 2. In such a stochastic scenario, the selection differential remains 0.6, but the opportunity for sexual selection is now 1.6. In this stochastic case, the variation in mating success has increased because there is now variation in mating success among trait‐bearing males. This variation in mating success among males that have the preferred trait in this scenario is due to chance

In the present study, we used a mathematical framework to begin to quantify the relative contribution of chance versus sexual selection to variation in mating success across biological scenarios of interest. We focus on a basic source of chance in our study: the chance that arises due to the fact that mate numbers must be integers (i.e., the chance mating success that arises because you cannot have a fraction of a mate; Figure 1). Even when sexual selection is strong and certain males are able to monopolize many female mates because of their traits, chance variation in mating success is inevitable because the number of mates per individual is a discrete number and this alone creates chance variation in mating success (Figure 1; (Jennions et al., 2012)). Previous work suggests that the fact that mating success must be a discrete number affects how sexual selection covaries with the operational sex ratio (i.e., the ratio of males to females that are prepared to mate at a given time and location in a population, OSR) in some cases (Jennions et al., 2012). Specifically, as the number of potential mates per individual decreases (i.e., as the OSR becomes more biased), variation in mating success increases, but the strength of sexual selection remains constant because the effect of chance on mating success increases when there are fewer potential mates per individual. In other words, the effect of chance varies systematically with potential mating success and with OSR (Jennions et al., 2012). How the effect of chance covaries with other factors of interest, and whether chance and OSR always covary, remains unknown. Here, we explore the relative contributions of chance and sexual selection to variation in mating success across scenarios that vary in mating dynamics. In doing so, we generate a priori predictions of the expected strength of sexual selection versus one source of chance (i.e., the chance that occurs because mate numbers must be an integer) across biological scenarios of interest.

## 2 METHODS

### 2.1 Model scenarios

We used a numerical model to illustrate the relationship between chance, mating success, and sexual selection. For simplicity, we assumed that sexual selection acts on a male trait and that males either have or do not have the preferred trait (i.e., the trait is binary). Such a trait could be thought of as a trait that is preferred in mate choice, a trait that allows males to access or monopolize females, or a trait that allows males to detect and locate females better than males who lack the trait (see, e.g., (Jennions et al., 2012)). We assume that the trait is selected for and that males must have the trait to be successful in mate acquisition. Males can mate multiply in the model, whereas females mate only once during a given reproductive episode, and as such, we focus on variation in male mating success and male sexual selection.

To begin to understand how chance can impact mating dynamics and sexual selection, we asked whether chance covaries with OSR and/or adult population size to influence mating success; that is, we asked whether OSR and/or adult population size affect the conditions under which chance will be relatively more or less important in determining mating success. We focused on OSR and adult population size because these two factors are thought to impact patterns of sexual selection across species (Emlen & Oring, 1977; Kokko & Rankin, 2006). Variation in OSR can be due to variation in the abundance of one sex and/or variation in the relative abundance of both sexes. As such, we varied OSR in two ways: In the model, OSR varied because 1) the absolute number of females present in the mating pool varied (Scenario 1) and 2) the relative number of males and females in the mating pool varied (Scenario 2). For both scenarios, we then considered two levels of adult population size, that is, small and large adult population sizes (Table 1). As mentioned previously, we assumed that males either have or lack a trait that is sexually selected. Because the abundance and relative distribution of preferred traits can vary substantially in nature, for each of the above scenarios, we considered cases in which (a) half, (b) three, or (c) two of all adult males had the preferred trait. Details of all model parameters are provided in Table 1. The specific OSR and adult population sizes considered were chosen to allow us to explore a range of biologically realistic OSRs and adult population sizes.

**TABLE 1:** Model Overview. To explore whether the operational sex ratio (OSR) and adult population size covary with chance to influence mating success, we considered three mating scenarios in which male and/or female numbers vary. For each scenario, we considered cases in which (i) 1/2 of all males have the preferred trait, (ii) 3 males have the preferred trait, or (iii) 2 males have the preferred trait. In the following table, we describe each scenario and the model parameters (male and female numbers, OSR, adult population size) considered. Additional model dynamics are as described in the text

Model scenario:	Model details:
**Scenario 1:** Variation in female numbers leads to changes in OSR when adult population size is (a) small versus (b) large when ½ of all males, 3 males, or 2 males have the preferred trait	**(a) Small Adult Population Size (14–34 adults)**: We consider an unbiased OSR (12:12 males:females, 24 adults), five male‐biased OSRs (males:females & adult population size = 12:2 & 14 adults, 12:4 & 16 adults, 12:6 & 18 adults, 12:8 & 20 adults, 12:10 & 22 adults), and five female‐biased OSRs (males:females & adult population size = 12:14 & 26 adults, 12:16 & 28 adults:, 12:18 & 30 adults, 12:20 & 32 adults, 12:22 & 34 adults) **(b) Large Adult Population Size (28–68 adults)**: We consider an unbiased (24:24 males:females, 48 adults) OSR, five male‐biased OSRs (males:females & adult population size = 24:4 & 28 adults, 24:8 & 32 adults, 24:12 & 36 adults, 24:16 & 40 adults, 24:20 & 44 adults), and five female‐biased OSRs (males:females & adult population size = 24:28 & 52 adults, 24:32 & 56 adults, 24:36 & 60 adults, 24:40 & 64 adults, 24:44 & 68 adults)
**Scenario 2:** Variation in relative adult numbers leads to changes in OSR when adult population size is (a) small versus (b) large when ½ of all males, 3 males, or 2 males have the preferred trait	**(a) Small Adult Population Size (24 adults)**: We consider an unbiased OSR (12:12 males:females), five male‐biased OSRs (22:2, 20:4, 18:6, 16:8, 14:10 males:females), and five female‐biased OSRs (10:14, 8:16, 6:18, 4:20, 2:22 males:females) **(b) Large Adult Population Size (48 adults)**: We consider an unbiased OSR (24:24 males:females), five male‐biased OSRs (44:4, 40:8, 36:12, 32:16, 28:20 males:females), and five female‐biased OSRs (20:28, 16:32, 12:36, 8:40, 4:44 males:females)

### 2.2 Calculation of mating success

For each of the above cases (see also Table 1), mating success was calculated in two ways: (a) deterministically, such that having the preferred trait was directly proportional to mating success and mate numbers were not limited to an integer (“no chance” scenario); and (b) stochastically, such that having the preferred trait was required for mating and therefore correlated with mating success, but mate number was restricted to an integer (“chance” scenario) (Figure 1). In doing so, we explored the effect of a simple source of stochasticity—the fact that mate number must be an integer—on mating success. For each scenario in the model, only males with the preferred trait could receive one or more female mates. Female mates were equitably assigned among the males who had the preferred trait in the deterministic scenario (see also Figure 1 for an example of deterministic mate assignment). As such, mating success in the deterministic scenario was calculated as follows:

(1) $$M_{+}=N_{+}/F$$

(2) $$M_{‐}=0$$

where *M_+_* is the mating success of each trait‐bearing male in the population, *N_+_* is the total number of trait‐bearing males in the population, *F* is the number of females in the population, and *M_−_* is the mating success of each non‐trait‐bearing male in the population.

In the stochastic scenario, female mates were assigned as equitably as possible among males with the preferred trait while restricting mating success to an integer value (see also Figure 1 for an example of stochastic mate assignment in which mating success is restricted to integer values). As such, when the number of females was evenly divisible among the number of males with the preferred trait, mating success of trait‐bearing males was calculated following Equation 1. When the number of females was not evenly divisible among the number of males with the preferred trait, Equation 1 could not be used, as it would lead to noninteger values of mating success. Instead, mating success for trait‐bearing males was calculated such that the total mating success of all trait‐bearing males in the population summed to *F*. Specifically, in all cases, all males with the preferred trait had an equal likelihood of receiving one or more female mates, but mating success for each male was restricted to an integer. For example, if there were five males with the preferred trait and two female mates, two males with the preferred trait were each assigned a mating success of one and the remaining three males were assigned a mating success of zero due to the lack of additional female mates. Likewise, if there were two males with the preferred trait and five female mates, one of the males was assigned a mating success of two and the other male was assigned a mating success of three. Since all males with the preferred trait were assumed to have an equal likelihood of receiving a mate, the variation in mating success among males with the preferred trait resulted from mating success being restricted to integer values in the stochastic cases. In the stochastic scenario, the mating success of non‐trait‐bearing males was zero (Equation 2).

### 2.3 Calculation of the strength of sexual selection, the opportunity for sexual selection, and variation in mating success due to chance

After assigning mates to males in each of the scenarios considered (Table 1) for both the deterministic and stochastic scenarios, we then calculated (a) the selection differential with respect to the preferred trait (*s*), which quantifies the strength of sexual selection on the phenotypic trait (Jones, 2009); and (b) the opportunity for sexual selection (*I_s_*), which is a standardized measure of variance in mating success in a population (Jones, 2009). The selection differential is directly proportional to the response to sexual selection that would be expected if (a) there is heritable variation associated with the trait and (b) mating success is correlated with reproductive success. Specifically, the selection differential was calculated for each scenario as follows:

(3) $$s=cov\left(\text{male trait value, relative mating success}\right)$$

where male trait value is the trait value (0 or 1) of each male and relative mating success is the relative mating success of each male in a population (i.e., the mating success of each male in the population divided by the mean male mating success in that population, (Jones, 2009)). In each scenario, and for the deterministic and stochastic cases, the opportunity for sexual selection was calculated as follows:

(4) $$I_{s}=V_{M}/\bar{M^{2}}$$

where *V_M_* is the variation in male mating success and $\bar{M}$ is mean male mating success within a population. Given that the opportunity for sexual selection is a standardized measure of variance in mating success, the variation in mating success that was due to chance was then quantified as the difference in the opportunity for sexual selection associated with the stochastic scenario and the opportunity for sexual selection associated with the deterministic scenario, such that:

(5) $$\text{Variance in mating success due to chance}=I_{s(Stochastic)}‐I_{s(deterministic)}$$

This measure allowed us to determine how much variation in mating success is caused by chance for each mating situation; that is, the variation in mating success due to chance quantifies the variation in mating success that, in our model, stems from the fact that mate number is an integer under the stochastic scenarios.

**FIGURE 2:**
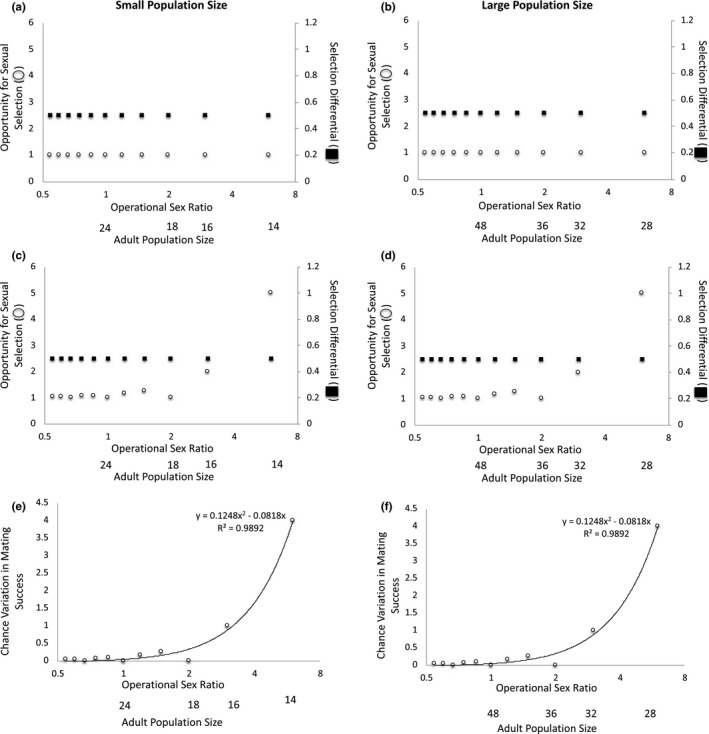
The operational sex ratio (OSR) and adult population size change as the number of females available to mate varies when half of all males present have the preferred trait. When mating is deterministic at (a) small and (b) large adult population sizes, the opportunity for sexual selection and the selection differential are invariant across OSRs. When mating is stochastic at (c) small and (d) large adult population sizes, the opportunity for sexual selection increases as OSR increases, but the selection differential remains constant across OSRs. Because the selection differential remains constant even as variation in mating success increases under the stochastic scenario, the magnitude of the variation in mating success that is due to chance increases as OSR increases at both (e) small and (f) large adult population sizes. Note: The x‐axis is presented as log_2_(OSR) to make low‐OSR data points visible

**FIGURE 3:**
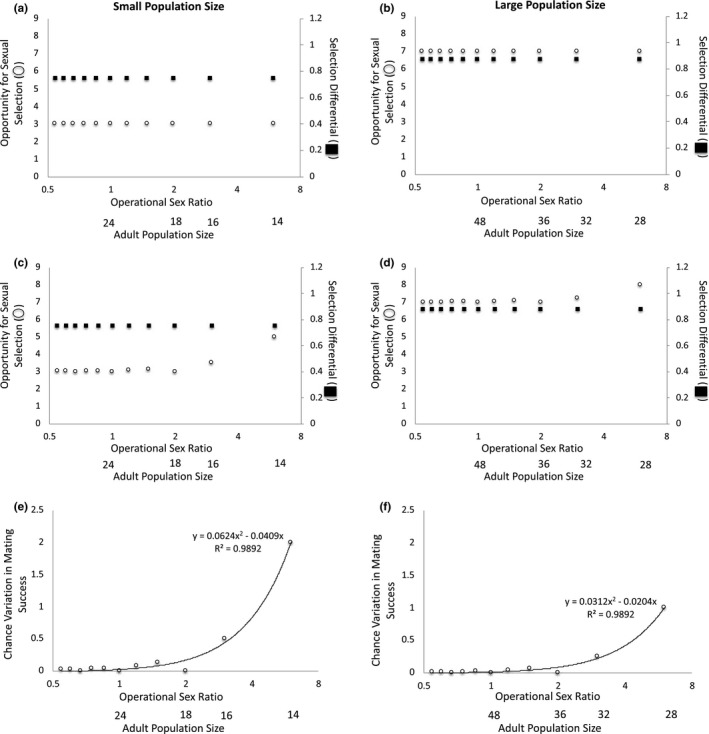
The operational sex ratio (OSR) and adult population size change as the number of females available to mate varies when three males have the preferred trait. When mating is deterministic at (a) small and (b) large adult population sizes, the opportunity for sexual selection and the selection differential are invariant across OSRs. When mating is stochastic at (c) small and (d) large adult population sizes, the opportunity for sexual selection increases as OSR increases, but the selection differential remains constant across OSRs. Because the selection differential remains constant even as variation in mating success increases under the stochastic scenario, the magnitude of the variation in mating success that is due to chance increases as OSR increases at both (e) small and (f) large adult population sizes. In addition, the proportion of variation in mating success that is due to chance is greater when adult population size is (e) small versus (f) large. Note: The x‐axis is presented as log_2_(OSR) to make low‐OSR data points visible

**FIGURE 4:**
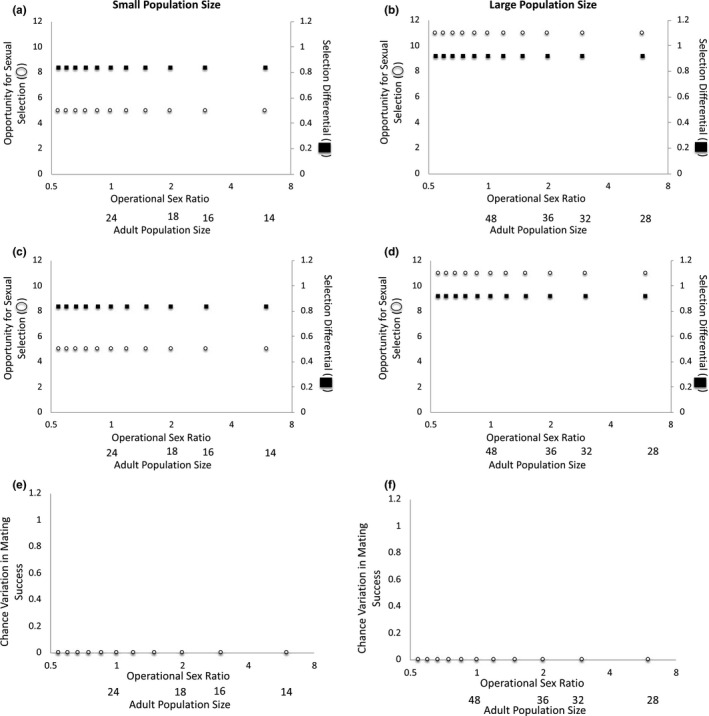
The operational sex ratio (OSR) and adult population size change as the number of females available to mate varies when two males have the preferred trait. When mating is deterministic at (a) small and (b) large adult population sizes and when mating is stochastic at (c) small and (d) large adult population sizes, the opportunity for sexual selection and the selection differential are invariant across OSRs. At both (e) small and (f) large adult population sizes, the magnitude of the variation in mating success that is due to chance is zero. Note: The x‐axis is presented as log_2_(OSR) to make low‐OSR data points visible

**FIGURE 5:**
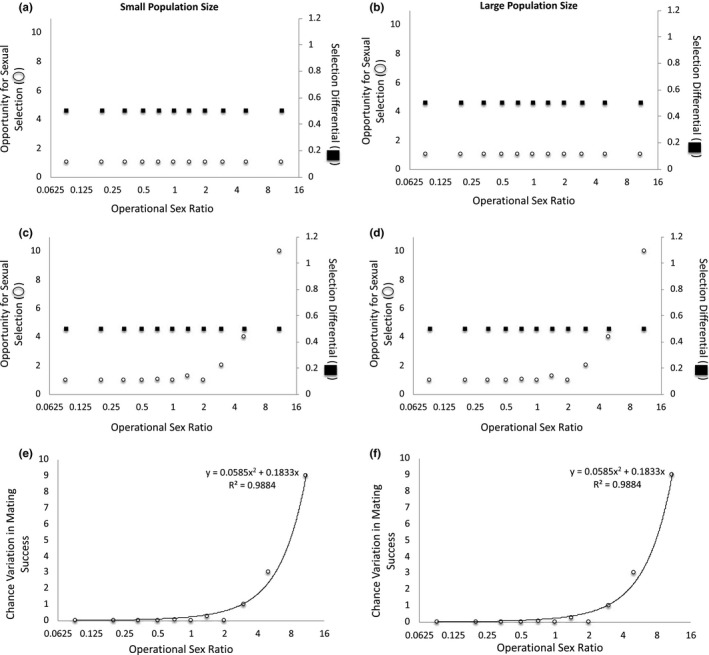
The operational sex ratio (OSR) changes as the relative number of males and females available to mate varies when half of all males present have the preferred trait. When mating is deterministic at (a) small and (b) large adult population sizes, the opportunity for sexual selection and the selection differential are invariant across OSRs. When mating is stochastic at (c) small and (d) large adult population sizes, the opportunity for sexual selection increases as OSR increases, but the selection differential remains constant across OSRs. Because the selection differential remains constant even as variation in mating success increases under the stochastic scenario, the magnitude of the variation in mating success that is due to chance increases as OSR increases at both (e) small and (f) large adult population sizes. Note: The x‐axis is presented as log_2_(OSR) to make low‐OSR data points visible

**FIGURE 6:**
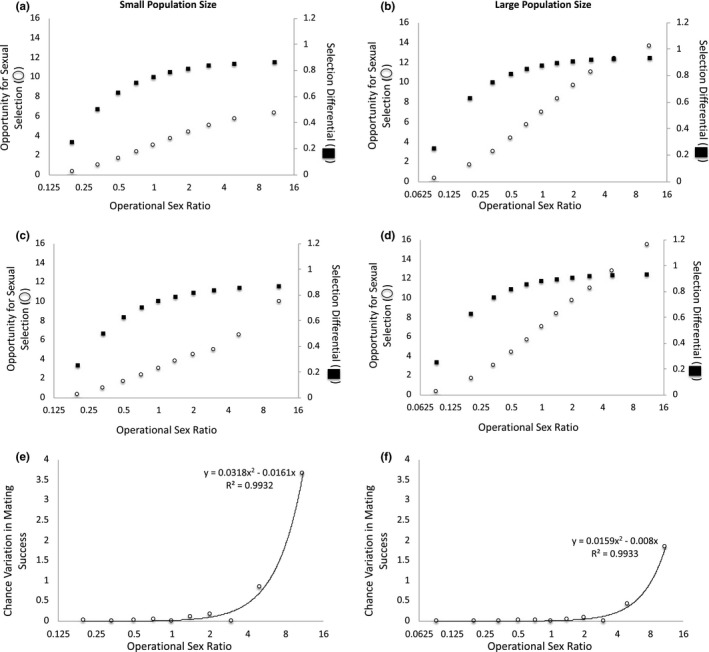
The operational sex ratio (OSR) changes as the relative number of males and females available to mate varies when three males have the preferred trait. When mating is deterministic at (a) small and (b) large adult population sizes and when mating is stochastic at (c) small and (d) large adult population sizes, the opportunity for sexual selection and the selection differential increase as OSR increases, although the magnitude of the increase in the opportunity for sexual selection is greater when mating is stochastic versus deterministic (c versus a and d versus b, open circles). The magnitude of the variation in mating success that is due to chance increases as OSR increases at both (e) small and (f) large adult population sizes, and the proportion of variation in mating success that is due to chance is greater when adult population size is (e) small versus (f) large. Note: The x‐axis is presented as log_2_(OSR) to make low‐OSR data points visible

**FIGURE 7:**
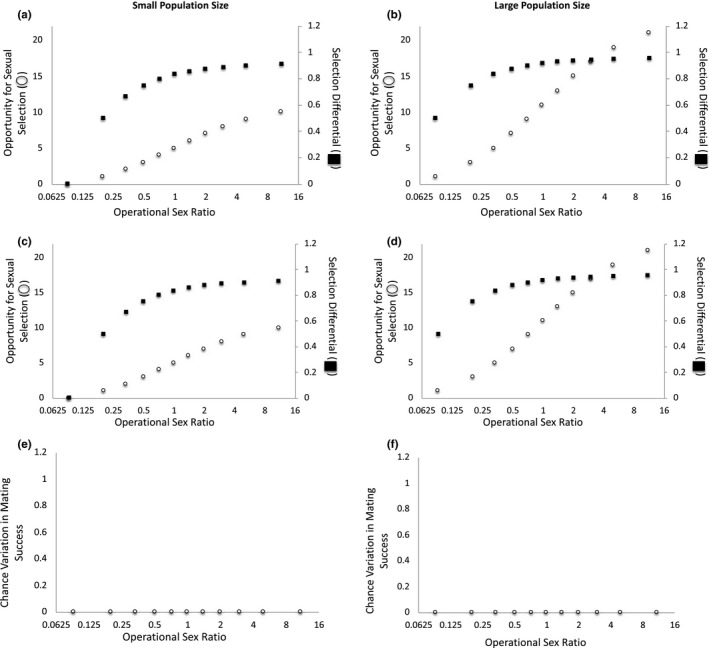
The operational sex ratio (OSR) changes as the relative number of males and females available to mate varies when two males have the preferred trait. When mating is deterministic at (a) small and (b) large adult population sizes and when mating is stochastic at (c) small and (d) large adult population sizes, the opportunity for sexual selection and the selection differential increase as OSR increases. At both (e) small and (f) large adult population sizes, the magnitude of the variation in mating success that is due to chance is zero. Note: The x‐axis is presented as log_2_(OSR) to make low‐OSR data points visible

For each scenario (scenarios 1–2), and at each adult population size and under each selection regime (Table 1), we then examined the relationship between the strength of sexual selection and the opportunity for sexual selection and the variation due to chance across OSRs and at both adult population sizes (Figures 2, 3, 4, 5, 6, 7). This allowed us to (a) identify the conditions under which adult population size, OSR, and selection regime affected the proportion of variation in mating success that is due to chance; and (b) explore the relative contribution of sexual selection versus chance in determining mating success across scenarios.

Based on previous research (Jennions et al., 2012), we expected that the effect of chance on mating success would covary with OSR. Additionally, we expected that chance would be relatively less important in determining mating success when sexual selection was relatively strong, which is expected to occur when relatively few males monopolize female mates. However, we had no a priori predictions of whether the effect of chance on sexual selection would covary with adult population size or whether adult population size, OSR, and/or the number of trait‐bearing males might interact to influence the effect of chance on mating success. Sexual selection will be strongest when one or a few males monopolize female mates because of their trait value. Thus, we hypothesized that the strength of sexual selection (i.e., the selection differential) would, on average, be greatest when the number of trait‐bearing males was relatively small.

## 3 RESULTS

### 3.1 Scenario 1: Variation in female abundance creates variation in OSR

In Scenario 1, we considered the case in which OSR varies because of differences in the number of females in the mating pool. In this case, OSR covaries with adult population size. When half of all males have the preferred mating trait, the opportunity for sexual selection and the strength of sexual selection remain constant across OSRs at small and large adult population sizes when mating is deterministic (Figure 2a–b). However, if we account for the fact that mate number must be an integer (i.e., if mating involves some chance), the opportunity for sexual selection increases as the OSR increases (i.e., becomes more male‐biased)—that is, there is greater variation in mating success as OSR becomes more male‐biased—but the strength of sexual selection on the male trait remains constant across OSRs at both small and large adult population sizes (Figure 2c–d). This pattern is explained by the fact that when stochasticity is accounted for, the number of males that remain unmated increases as the OSR becomes more male‐biased. That is, at male‐biased OSRs, some trait‐bearing males are unable to obtain a mate due to chance when mate number is restricted to an integer because there are fewer females than there are trait‐bearing males. This, in turn, creates greater variation in male mating success at male‐biased OSRs, and chance therefore becomes relatively more important in determining mating success as the OSR becomes more male‐biased. Indeed, in this case, the magnitude of the variation in mating success that is due to chance increases as OSR becomes more male‐biased (Figure 2e–f), and this effect of OSR on the amount of variation in mating success that is due to chance is the same at small and large adult population sizes (Figure 2e–f). Thus, when variation in female number creates variation in OSR and half of the males in a population have the preferred trait, the effect of chance on mating success covaries with OSR but not adult population size to determine mating success.

When half of the males have the preferred trait in this scenario, the selection differential is invariant across OSRs and population sizes (*s = *0.5 in all cases) (Table 3). If the trait is heritable and mating success is correlated with reproductive success, we would expect the evolutionary response to sexual selection to be the same across OSRs and population sizes in this case. That is, under these conditions, and despite the greater variance in mating success under the stochastic scenario, we would not expect OSR or adult population size to affect sexual selection.

When (a) OSR varies because of differences in the number of females in the mating pool and (b) three males have the preferred mating trait, the opportunity for sexual selection and the selection differential remain constant across OSR levels at small and large adult population sizes when mating is deterministic (Figure 3a–b). When mating is stochastic, increases in OSR are associated with increases in the opportunity for sexual selection, but the selection differential remains constant across OSRs (Figure 3c–d). As a result, the variation in mating success that is due to chance increases as OSR increases (Figure 3e–f). This qualitative pattern is similar at small and large population sizes, but the magnitude of variation that is due to chance is greater when adult population size is small versus large (Figure 3e–f). In this case, adult population size influences the variation in mating success that is due to chance because when population size is relatively large, a smaller proportion of males have the preferred trait and monopolize female mates (and hence, selection is greater) relative to the case in which adult population size is small (Figure 3a,c versus b,d). In turn, when selection is greater, a relatively smaller proportion of the variation in mating success is due to chance. In summary, when variation in female number creates variation in OSR and three males have the preferred trait, the effect of chance on mating success covaries with both OSR and adult population size.

When three males have the preferred trait, the strength of sexual selection is greater when population size is large (*s = *0.875) versus small (*s = *0.75) (Table 3). This difference in the strength of sexual selection is consistent across OSRs and occurs because (a) a smaller proportion of males monopolize female mates and (b) a greater proportion of males remain unmated when population size is relatively large versus small. If the trait is heritable and mating success correlates with reproductive success, we would thus expect the evolutionary response to sexual selection to be greater when population size is relatively large. In contrast, sexual selection and the evolutionary response to sexual selection are expected to be the same across OSRs for a given level of population size (small or large) under this scenario (Table 3). As such, under these conditions, we would expect adult population size, but not OSR, to influence the strength of sexual selection. In addition, the strength of sexual selection is greater when three males have the preferred trait in comparison with the previous case in which half of all males have the preferred trait (*s = *0.5 when half of all males have the trait). As such, assuming that the trait is heritable and mating success is correlated with reproductive success, we would expect a greater evolutionary response to sexual selection when three males have the preferred trait in comparison with the case in which half of all males have the preferred trait (Figure 3 versus 2).

When (a) OSR varies because of differences in the number of females in the mating pool and (b) two males have the preferred mating trait and monopolize all female mates, the opportunity for sexual selection and the selection differential remain constant across OSR levels at small and large adult population sizes when mating is deterministic (Figure 4a–b) and when mating is stochastic (Figure 4c–d). In this case, all variation in mating success is due to selection and no variation in mating success is due to chance at either small or large adult population sizes (Figure 4e–f). Unlike the case above in which three males have the preferred trait, in this case, all variation in mating success is due to selection (and not chance) because female mates are evenly divisible among the trait‐bearing males in the population. This suggests that whether chance variation in mating success necessarily exists in a population will depend on whether female mates can be equitably spread among trait‐bearing males. When female mates cannot be evenly divided among trait‐bearing males (as in the scenario above in which three males had the preferred trait), some chance variation in mating success is inevitable; in contrast, when female mates are evenly divisible among trait‐bearing males (as in the scenario in which two males have the preferred trait), chance variation in mating success that stems from the fact that mating success must be an integer will not occur. Importantly, these patterns suggest that the number of trait‐bearing males and the adult population size will interact to determine whether chance variation in mating success occurs due to mate numbers being restricted to an integer value.

When two males have the preferred trait, the selection differential is greater at large (*s = *0.917) versus small (*s = *0.833) population sizes. Within a given population size level (small versus large), the selection differential is invariant across OSRs. Thus, under these conditions, we would expect population size but not OSR to influence the strength of sexual selection (Table 3). As such, if the trait is heritable and if mating success is correlated with reproductive success, we would expect a greater response to sexual selection at relatively large population sizes. As in the previous case, this occurs because a smaller proportion of males have the trait and monopolize female mates, and a larger proportion of males remain unmated, when population size is relatively large versus small.

### 3.2 Scenario 2: OSR changes as relative male and female numbers change

In Scenario 2, the OSR changes as the relative number of males and females in the mating pool varies. In this case, when half of the males have the preferred trait, the opportunity for sexual selection and the selection differential are constant across OSRs when mating is deterministic at both small and large adult population sizes (Figure 5a–b). However, when we account for the stochasticity that occurs because mate number must be an integer, the opportunity for sexual selection increases as OSR increases, but the selection differential remains constant across OSRs at both small and large adult population sizes (Figure 5c–d). In this case, the magnitude of the variation in mating success that is due to chance increases as OSR increases (Figure 5e–f). This effect of OSR on the amount of variation in mating success that is due to chance is the same at small and large adult population sizes and occurs because more trait‐bearing males, by chance, remain unmated when there are fewer female mates per male in the population. Thus, when variation in relative male and female abundance creates variation in OSR and half of all males have the preferred trait, the effect of chance on mating success covaries with OSR but not adult population size.

When half of the males have the preferred trait in this scenario, the selection differential is invariant across OSRs and population sizes (*s = *0.5 in all cases). Under the conditions of this case, we would not expect OSR or population size to affect the strength of sexual selection. Assuming that the trait is heritable and mating and reproductive success are correlated, we would expect the evolutionary response to sexual selection to be the same across OSRs and population sizes under the conditions of this scenario (Table 3).

When (a) the OSR changes as the relative number of males and females in the mating pool varies and (b) three males have the preferred trait, the opportunity for sexual selection and the selection differential increase as the OSR becomes more male‐biased for both deterministic and stochastic mating regardless of whether adult population size is small versus large (Figure 6a–d). However, the magnitude of the increase in the opportunity for sexual selection is greater when mating is stochastic versus deterministic (Figure 6c versus a and d versus b, open circles). As such, the variation in mating success that is due to chance increases as the OSR becomes more male‐biased (Figure 6e–f). This occurs because there are relatively few females per trait‐bearing males at more male‐biased OSRs. As a result, some trait‐bearing males remain unmated due to chance at highly male‐biased OSRs. Additionally, chance creates greater variation in mating success when adult population size is small versus large (Figure 6e–f). In this case, adult population size influences the variation in mating success that is due to chance because a smaller proportion of males have the preferred trait and monopolize female mates (and hence, selection is greater) when population size is relatively large relative to the case in which adult population size is small. In summary, when the OSR changes as the relative number of males and females in the mating pool changes and three males have the preferred trait, the effect of chance on mating success covaries with both OSR and adult population size.

When three males have the preferred trait, selection increases as the OSR becomes more male‐biased (Figure 6c and d) and is on average stronger when population size is large (Figure 6d, large population range in *s* = 0.25–0.932) versus small (Figure 6c, small population range in *s* = 0.25–0.864). The strength of sexual selection, and the expected evolutionary response to sexual selection, is therefore expected to be greater at more male‐biased OSRs and when adult population size is relatively large (Table 3), as these are the conditions under which (a) a relatively small number of males monopolize female mates and (b) a relatively large number of males remain unmated.

When (a) the OSR changes as the relative number of males and females in the mating pool varies and (b) two males have the preferred trait, the opportunity for sexual selection and the selection differential increase as OSR increases at small and large adult population sizes when mating is deterministic (Figure 7a–b) and when mating is stochastic (Figure 7c–d). In this case, all variation in mating success is due to selection and no variation in mating success is due to chance at either small or large adult population sizes (Figure 7e–f). As in Scenario 1, this pattern occurs because the number of female mates is evenly divisible among the trait‐bearing males in the population and relatively few males monopolize all female mates. If the number of female mates was not evenly divisible among the trait‐bearing males (as in the case above in which three males have the preferred trait), chance variation in mating success would exist.

When two males have the preferred trait in this scenario, selection increases as OSR increases for each population size level (Figure 7c and d) and is on average greater when population size is large (Figure 7d, large population range in *s* = 0.5–0.955) versus small (Figure 7c, small population range in *s* = 0–0.91). As in the case in which three males have the preferred trait, the strength of sexual selection, and the expected evolutionary response to sexual selection, is thus expected to be greater at more male‐biased OSRs and at relatively large population sizes (Table 3) because these are the conditions under which (a) a relatively small number of males monopolize female mates and (b) a relatively large number of males remain unmated.

## 4 DISCUSSION

Here, we have illustrated that the importance of chance in determining mating success can, in some cases, covary with biological factors of interest such as OSR and adult population size. However, whether OSR and adult population size covary with chance to impact mating success will depend on (a) the number of trait‐bearing males in the population and (b) male and female abundance in the mating pool (Table 2). When a small number of males have the preferred trait, mate monopolization is strong, and female mates can be equitably divided among trait‐bearing males, all variation in mating success will be due to sexual selection regardless of OSR or adult population size (Table 2; Figures 4, 7c–f). In contrast, when half of all males have the preferred trait (i.e., when mate monopolization is not strong), chance variation in mating success increases as OSR increases (Table 2; Figures 2 and 5). A similar pattern is observed when a small number of males have the preferred trait and female mates cannot be equitably divided among trait‐bearing males: In this case, some males remain unmated simply due to the fact that mate number must be an integer, and this creates chance variation in mating success, particularly at more male‐biased OSRs (Table 2; Figures 3 and 6). In these cases, chance contributes relatively more to the overall variation in mating success at greater OSRs because an increasing number of males remain unmated even if they have the preferred trait as the sex ratio becomes more male‐biased.

**TABLE 2:** A summary of the effect of OSR, adult population size, and chance on mating success. Modeling scenarios are as described in the text

Scenarios	Does OSR covary with chance to influence mating success?	Does adult population size covary with chance to influence mating success?
Scenario 1: Variation in female abundance leads to variation in OSR and (i) ½ of all males, (ii) 3 males, or (iii) 2 males have the preferred trait	(i) Yes, variation in mating success due to chance increases as OSR increases (Figure 2c–f)	(i) No, variation in mating success due to chance is the same when adult population size is small versus large (Figure 2e versus f)
	(ii) Yes, variation in mating success due to chance increases as OSR increases (Figure 3c–f)	(ii) Yes, variation in mating success due to chance is greater when adult population size is small versus large (Figure 3e versus f)
	(iii) No, across OSRs, variation in mating success is caused only by selection (Figure 4c–f)	(iii) No, across adult population size levels, variation in mating success is caused only by selection (Figure 4e versus f)
Scenario 2: Variation in the relative abundance of males and females leads to variation in OSR and (i) ½ of all males, (ii) 3 males, or (iii) 2 males have the preferred trait	(i) Yes, variation in mating success due to chance increases as OSR increases (Figure 5c–f)	(i) No, variation in mating success due to chance is the same when adult population size is small versus large (Figure 5e versus f)
	(ii) Yes, variation in mating success due to chance increases as OSR increases (Figure 6c–f)	(ii) Yes, variation in mating success due to chance is greater when adult population size is small versus large (Figure 6e versus f)
	(iii) No, across OSRs, variation in mating success is caused only by selection (Figure 7c–f)	(iii) No, across adult population size levels, variation in mating success is caused only by selection (Figure 7e versus f)

In addition, in some cases, chance becomes increasingly more important at determining mating success at smaller population sizes. When a small, fixed number of males have the preferred trait and female mates cannot be equitably divided among trait‐bearing males, chance contributes relatively more to variation in mating success when population size is small versus large (Table 2; Figures 3 and 6). This occurs because (a) due to chance, not all males with a preferred trait will receive the same number of mates when the number of females is not equally divisible among trait‐bearing males; and (b) when a fixed number of males have the preferred trait, sexual selection is greater when the population size is relatively large since more males remain unmated (and hence, a relatively greater proportion of the variation in mating success is due to sexual selection rather than chance). In contrast, if a small number of males have the preferred trait and female mates can be equitably divided among trait‐bearing males, all variation in mating success is due to sexual selection at both large and small population sizes (Figures 4 and 7). Similarly, if half of all males have the preferred trait, the relative contribution of chance versus sexual selection to variation in mating success is the same at small and large population sizes (Figures 2 and 5).

Fully understanding the operation of mate acquisition necessitates an understanding of the relative contribution of chance versus sexual selection to variation in mating success. In general, our results suggest that, in some cases, chance can covary with OSR and/or adult population size to influence the relative amount of variation in mating success that is due sexual selection. Our finding that the relative effect of chance on mating success can, under some conditions, depend on OSR and adult population size expands upon previous work (Jennions et al., 2012) and is consistent with the expectation that chance will influence mating success even when sexual selection is operating (Hubbell & Johnson, 1987). Indeed, several previous studies have noted that chance can influence mating success and sexual selection acting on traits (Gowaty & Hubbell, 2009, 2013; Sutherland, 1985, 1987). Across the scenarios considered here, our results also suggest that the importance of chance in creating variation in mating success will depend heavily on (a) the number of trait‐bearing males, (b) the number of adult males and females in the population, and (c) whether the number of female mates is evenly divisible among trait‐bearing males. This is a biologically relevant finding, as male and female mate numbers, as well as the number of individuals with a preferred trait, are expected to vary through space and time within populations. It seems likely that, in many cases, female mate numbers will not be equitably divisible among males with a preferred trait in nature. Importantly, even if other sources of chance create variation in mating dynamics (discussed further below), the fact that mate numbers must be an integer is expected to create some baseline chance variation in mating success in many populations.

In addition, understanding that chance can, under some conditions, become increasingly more important in determining mating success and reduce the strength of sexual selection relative to its maximum potential at greater OSRs or smaller adult population size levels alters our expectations of when sexual selection is expected to be strong versus weak. For example, classic sexual selection theory predicts that the strength of sexual selection will increase as OSR increases (Emlen & Oring, 1977). However, the findings of the present study and of previous work (Jennions et al., 2012) suggest that the strength of sexual selection should not always be expected to increase as OSR increases. In particular, when variation in female abundance leads to variation in OSR, our findings suggest that more male‐biased OSRs will not lead to stronger sexual selection (Table 3). When variation in the relative abundance of males and females leads to variation in OSR, more male‐biased OSRs are only expected to lead to stronger sexual selection when a small number of males have the preferred trait (Table 3). Further, our findings suggest that the strength of sexual selection can be sensitive to adult population size in some cases. When a relatively small, fixed number of males have the preferred trait, sexual selection tends to be stronger at relatively large population sizes (Table 3).

**TABLE 3:** A summary of the effect of the operational sex ratio (OSR) and adult population size on the strength of sexual selection. Modeling scenarios are as described in the text

Scenarios	Does OSR affect the strength of sexual selection?	Does adult population size affect the strength of sexual selection?
Scenario 1: Variation in female abundance leads to variation in OSR and (i) ½ of all males, (ii) 3 males, or (iii) 2 males have the preferred trait	(i) No, the selection differential is invariant across OSRs (Figure 2c–d)	(i) No, the selection differential is the same at small and large population sizes (Figure 2c–d)
	(ii) No, the selection differential is invariant across OSRs (Figure 3c–d)	(ii) Yes, the selection differential is greater when population size is large versus small (Figure 3c–d)
	(iii) No, the selection differential is invariant across OSRs (Figure 4c–d)	(iii) Yes, the selection differential is greater when population size is large versus small (Figure 4c–d)
Scenario 2: Variation in the relative abundance of males and females leads to variation in OSR and (i) ½ of all males, (ii) 3 males, or (iii) 2 males have the preferred trait	(i) No, the selection differential is invariant across OSRs (Figure 5c–d)	(i) No, the selection differential is the same at small and large population sizes (Figure 5c–d)
	(ii) Yes, on average, the selection differential increases as OSR increases (Figure 6c–d)	(ii) Yes, on average, the selection differential is greater at large versus small population sizes (Figure 6c–d)
	(iii) Yes, on average, the selection differential increases as OSR increases (Figure 7c–d)	(iii) Yes, on average, the selection differential is greater at large versus small population sizes (Figure 7c–d)

Importantly, in the present study, we have considered only one basic source of chance variation in mating success: the variation that arises due to the fact that mate number must be an integer. Chance variation in mating success is likely additionally due to a range of other factors, including imprecise mate sampling and assessment. In the future, it will be important to explore how other sources of stochasticity contribute to variation in mating success across OSRs and population sizes. In particular, understanding the additive and relative contribution of (a) mate number being constrained to an integer and (b) imprecise mate sampling and assessment to stochastic variation in mating success will be key for determining if and when chance is likely to have substantial effects on mating dynamics. In addition, it will also be interesting to empirically explore how the effect of chance on mating success changes across OSRs and densities and through time. For example, an empirical study could examine the relationship between variation in mating success and the strength of sexual selection on a trait across OSRs and adult population sizes to determine whether the data are consistent with the prediction that chance will become more important in determining mating success at highly male‐biased OSRs and smaller population sizes. More generally, in the future, fully understanding the operation of sexual selection and mating systems will require that we determine the relative contribution of sexual selection, natural selection, and chance to variation in mating and reproductive success.

## CONFLICTS OF INTEREST

The authors have no conflicts of interest.

## AUTHOR CONTRIBUTION

**Hope Klug:** Conceptualization (lead); Data curation (equal); Formal analysis (equal); Funding acquisition (lead); Investigation (equal); Methodology (lead); Supervision (lead); Writing‐original draft (equal); Writing‐review & editing (lead). **Libby Stone:** Data curation (equal); Formal analysis (equal); Investigation (equal); Writing‐original draft (equal).

## Data Availability

All data are available in Dryad (https://doi.org/10.5061/dryad.0rxwdbrzz).
